# The role of the respiratory microbiome in asthma

**DOI:** 10.3389/falgy.2023.1120999

**Published:** 2023-05-30

**Authors:** Christina D. Campbell, Margaret Gleeson, Imran Sulaiman

**Affiliations:** ^1^ Department of Respiratory Medicine, Royal College of Surgeons in Ireland, Dublin, Ireland; ^2^ Department of Respiratory Medicine, Beaumont Hospital, Dublin, Ireland

**Keywords:** asthma, microbiome, respiratory microbiome, biologic therapy, childhood asthma

## Abstract

Asthma is a common airways disease and the human microbiome plays an increasingly recognised role in asthma pathogenesis. Furthermore, the respiratory microbiome varies with asthma phenotype, endotype and disease severity. Consequently, asthma therapies have a direct effect on the respiratory microbiome. Newer biological therapies have led to a significant paradigm shift in how we treat refractory Type 2 high asthma. While airway inflammation is the generally accepted mechanism of action of all asthma therapies, including both inhaled and systemic therapies, there is evidence to suggest that they may also alter the microbiome to create a more functionally balanced airway microenvironment while also influencing airway inflammation directly. This downregulated inflammatory cascade seen biochemically, and reflected in improved clinical outcomes, supports the hypothesis that biological therapies may in fact affect the microbiome-host immune system dynamic and thus represent a therapeutic target for exacerbations and disease control.

## Introduction

Asthma is a heterogeneous disease, characterised by chronic airway inflammation, airway hyper-responsiveness and variable airflow obstruction. Symptoms include wheeze, dyspnoea, chest tightness and cough, which vary in intensity over time. Asthma is diagnosed with a history of classic symptoms coupled with evidence of variable airflow obstruction. Individuals with asthma can experience intermittent exacerbations of their disease, with increasing symptoms and airflow obstruction. Importantly, asthma is a common condition, with increasing prevalence worldwide. The Global Initiative for Asthma (GINA) states asthma affects 1%–8% of the global population (1) and there is an increasing prevalence worldwide (2), therefore understanding disease mechanisms is crucial in furthering the management and development of new treatments. With newer investigative tools, the microbiome has become of particular interest in the study of asthma.

The respiratory microbiome entails the collected genome of all micro-organisms inhabiting the respiratory tract. This includes all bacteria, viruses, bacteriophages, fungi and archaea. Microbiota refers to the community of micro-organisms, although the terms are often used interchangeably in the literature. Previously, the majority of research has focused on bacteria present, however next generation sequencing has expanded our ability to also study viruses, bacteriophages, fungi and archaea.

Development of 16S rRNA sequencing in the 1970s allowed assessment of bacterial phylogeny (3). A small subunit of the ribosomal gene, referred to as 16S, is transcribed. This gene is targeted as it is a conserved component of the transcriptional apparatus of all DNA based lifeforms. The gene is made up of nine constant and nine hypervariable regions. It is the hypervariable regions which allow identification of different bacteria. Next generation sequencing of the 16S rRNA subunit using gene amplicon sequencing lets investigators analyse bacterial composition of many samples simultaneously, at low cost.

Fungal Interval Transcribed Spacer (ITS) Sequencing is a similar approach for assessment of fungal phylogeny. Here, next generation sequencing of the ITS region of the nuclear ribosomal RNA (rRNA) is sequenced, allowing quantification of abundance of fungi present (4).

Newer studies utilising whole genome sequencing, or meta-genomics allows sequencing of all genetic material in the sample. The allows assessment of the abundance of bacteria, viruses, fungi and archaea simultaneously (5). RNA metatranscriptome sequencing then allows assessment of total microbial-RNA in a sample (5), revealing information on active gene expression and insights into the functional potential of the microbiome.

### Respiratory microbiome in health

The respiratory microbiome is formed at birth, with rapid development in the first weeks of life, with evidence that it is essential for normal lung development (6). The composition of the microbiome is affected by many differing factors, including breast feeding, antibiotic use, presence of siblings, vaccination status, environmental factors and childhood infections (7). Once established, the respiratory microbiome exists as a continuum, with decreasing microbial density down the respiratory tract and varying in composition by location (8).

The upper airway microbiome is complex, with composition differing greatly between the various locations, including the hard and soft tissues, saliva, tongue and plaque. In short, the healthy upper airway microbiome is dominated by *Streptococci*, followed by *Neisseria*, *Prevotella*, *Rothia*, and *Haemophilus* (4).

Similarly, the upper airway virome and mycome will vary. In general, the virome is abundant with the Herpesviridae family and includes several phages of the Siphoviridae, Myoviridae and Podoviridae families, which are directed against the most abundant bacterial species in the oral microbiome (9). The upper airway mycobiome is predominantly enriched with Candida and Saccharomicetale.

Moving to the lower respiratory tract, here the bacterial microbiome is composed of Bacteroidetes, Proteobacteria, Firmicutes and at genus level, *Streptococcus*, *Prevotella* and *Veillonella* (10). The virome mainly contains Anelloviridae, with herpes viruses and human papillomavirus also (11). The mycobiome is then made up of Ceriporia lacerate, Saccaromyces cerevisiae and Penicillium brevicompactum (12). It is important to appreciate that the lower respiratory microbiome is also transient, with microbes arriving by inhalation, micro-aspiration and direct mucosal spread from the upper airways, then cleared by the muco-ciliary system and innate immune system.

### Microbiome in childhood asthma

Childhood asthma is common; one in ten children will report asthma symptoms in the preceding year (13). It is generally preceded by acute viral wheezing in infancy, predominantly caused by rhinovirus and RSV (14). Multiple studies have demonstrated that acute wheezy episodes or incidences of bronchiolitis attributed to RSV in childhood is a risk factor for further asthma development (14, 15). The underlying mechanism is unclear, however altered immune response during acute lower respiratory illnesses, demonstrated by increased IgE and eosinophils was observed in children who subsequently developed persistent wheeze (16).

However, there is a growing body of evidence that bacterial pathogens detected by qualitative PCR during acute wheeze episodes, mainly *S. pneumoniae, H. influenzae* and *M. catarrhalis also* contribute to these severity of these events (17). The exact mechanism underling this connection is unclear, however, it is likely that children co-infected with RSV and viral pathogens experience increased airway inflammation (17). Additionally, asymptomatic detection of these pathogens in the nasopharynx in early life is associated more severe wheeze episodes in early childhood and the development of asthma by 5 years of age (18).

A long standing hypothesis in the pathogenesis of asthma relates to the hygiene hypothesis and exposure to irritants. Indeed, further exposure to irritants, such as pollen may increase symptoms in patients with asthma. The hygiene hypothesis postulates the protective effect of microbial exposures in early life on the development of allergy and asthma, and that lack of infections or exposure to potential pathogens during immune system maturation leads to greater risk of atopic diseases including asthma. This is supported by evidence, for example, that farm living is protective against asthma development, with further studies suggesting the presence of pets is also protective against asthma and allergy (19).

Furthermore, the presence of pathogens in the neonatal nasopharyngeal microbiome, such as *S. pneumoniae*, *M. catarrhalis* and *H. influenza*, as determined by traditional culture-dependent techniques, is associated with early childhood wheeze and the presence of asthma at the age of 5 years (18). This finding has been replicated in further studies utilising 16S rRNA gene sequencing, demonstrating a *Staphylococcus*-dominant upper respiratory tract microbiome in early childhood is associated with recurrent wheeze and persistent childhood asthma (20, 21).

The gut microbiome in childhood has a strong influence on the developing immune system, and in turn asthma, via the Gut-Lung Axis. Longitudinal childhood studies have demonstrated a strong link between childhood gut microbial dysbiosis and subsequent asthma development. In fact, multiple studies have demonstrated that an immature gut microbiota between 2 months to 1 year of life is associated with increased risk of asthma in later childhood (22–25), particularly in those with reduced abundance of *Akkermansia*, *Bifidobacterium*, and *Faecalibacterium* (22). Animal models and *in vivo* studies have suggested the importance of short chain fatty-acids (SCFAs), such as butyrate and propionate in the development of asthma. Butyrate contributes to the integrity of the epithelial gut barrier (26) and suppresses the production of proinflammatory mediators. Additionally, it has a direct effect on eosinophils, inhibiting eosinophil migration, adhesion to the endothelium and can induce eosinophil apoptosis (27). In these murine models, high fibre diets increase the abundance of Bacteroides and Bifidobacterium, which digest fibre to produce SCFA (28). Subsequently, mice fed high fibre diets have increased levels of circulating SCFA, both locally in the gastro-intestinal tract tract and systemically. The circulating SCFA then increase the haematopoiesis of dendritic cell precursors from bone marrow, which have a decreased tendency to activate Th 2 cells in the lung, ultimately decreasing airway inflammation and airway hyper-responsiveness (28). In human studies, children with a mature gut microbiome enriched with taxa capable of producing butyrate, and increased faecal butyrate have decreased rates of asthma in later life (25). Additionally, children who have an microbiome deplete in SCFA producing bacteria have an increased risk of asthma development (24).

Fungal exposures appear to influence asthma development, with evidence that childhood sensitisation to Alternaria is associated with later development of asthma (29). Another study found that children born during seasons with elevated ambient fungal sport concentrations is associated with wheeze in early life (30). Fungi within the gut microbiome also appear to have an effect on asthma development. Fungal dysbiosis in the gut microbiome is associated with bacterial dysbiosis and later asthma development (31). Again in murine models, antibiotic induced overgrowth of *Candidia parapsilosis* has been linked to airway inflammation (32). A separate study showing reduced abundance of *Lactobacillus* is also associated with fungal overgrowth and airway inflammation (33). Notably, *Lactobacillus* is a lactic acid producing bacteria, with established anti-fungal effects (34). Interestingly, germ-free mice and conventional mice with an established microbiome demonstrate differing inflammatory responses when exposed to a dysbiotic fungal microbiome, highlighting the complex nature of intra-kingdom interactions within the microbiome (35).

Archaea represent a little studied component of the human microbiome, however one study examining the gut microbiome in children demonstrated *M. stadtmanae* in stool samples was associated with a reduced risk of asthma development. The role of the virome in the gut microbiome and its effect on asthma remains to be discovered (36).

In asthma, there is clear evidence that receiving one or more antibiotic courses before 1 year of age is associated with a 50% increase in the risk of an asthma diagnosis (24, 37, 38). Additionally, from a large population based study in Canada, researchers were able to demonstrate a correlation with a decline in asthma incidence and large decreases in antibiotic prescription during infancy (24). Moreover, antibiotics have a direct effect on the gut microbiome and have been subsequently shown to affect a range of disease states (39). Antibiotics reduce species diversity, in turn altering metabolic activity and selecting for antibiotic resistant organisms such as *Clostridioides difficile* infection (39, 40). Importantly, depletion of SCFA-producing bacteria in the gut microbiome by antibiotic administration is also associated with increased rates of asthma development (24).

Naturally, these findings have led to an interest in the use of probiotics for asthma prevention or management. Several randomised control trials have examined the role of probiotics in asthma and other allergic diseases, with a range of bacterial strains, dosages, populations targeted and outcomes measured. Some have been positive; administration of *Lactobacillus paracasei* and *Lactobacillus fermentum* to children aged 6–18 years reduced asthma severity (41), and a separate study found a combination of *Ligilactobacillus salivarius* and *Bifidobacterium breve* reduced asthma exacerbation frequency (42). However, in another study, *Lactobacillus rhamnosus* administered in the first 6 months of life did not reduce incidence of asthma (43). Although some studies have demonstrated positive results, meta-analysis have so far failed to demonstrated an overall beneficial effect of probiotics (44) in the incidence or management of asthma,.

Asthma is a complex disease with many risk factors in early childhood, both environmental and genetic. Rich microbial exposures in childhood appear protective. Respiratory and gut microbial dysbiosis, reduced microbial diversity and pathogen exposure in childhood likely contribute to the development of asthma in later life. We will now discuss the lung microbiome, virome and mycome in established asthma, its role in asthma phenotypes, endotypes and various asthma treatments.

### Microbiome in adult asthma

The microbiota in asthma is diverse, varying with disease severity, clinical phenotype, current clinical status and underlying inflammation. The studies addressing the lung microbiome have employed a variety of sampling methods and diverse patient cohorts.

Importantly, the lung microbiome in asthma displays a higher bacterial burden than healthy controls, first demonstrated on bronchial brushings (45) and replicated on BAL and induced sputum (10, 45, 46). Some studies have found a reduction in commensal organisms, *Prevotella* and *Veillonella*, with an enrichment of Proteobacteria, mainly *Haemophilus*, *Neisseria* and *Moraxella* in mild asthma, in those not on inhaled corticosteroids (10, 45–47). However, it is important to appreciate that sampling site can influence study results. Induced sputum is frequently used as a surrogate for the lower respiratory microbiome, however sputum itself contains secretions from the lower and upper airways. A comparison study of bronchial brushing, induced sputum, oral wash and nasal brushings in adults with mild asthma demonstrated differing microbial composition within each sample. Similarity was greatest between bronchial brushing and induced sputum, however induced sputum still demonstrated a distinct microbiota with enrichment of oral bacteria (48).

Viruses play a central role in the development of asthma and exacerbations. Thus, the respiratory virome in adult asthma differs from that of healthy controls, with a predominance of Herpes viruses, of which Herpes Simplex Virus 1 and cytomegalovirus (CMV) make up the majority of the sputum virome. Both CMV and Ebstein-Bar Virus (EBV) abundance increases with asthma disease severity. Increased abundance of these micro-organisms is also negatively correlated with symptom burden and airway obstruction (49). Conversely, asthmatics also have decreased abundance of bacteriophages compared to healthy controls, predominantly consisting of a declining proportion of the *Streptococcus* phage. As bacteriophages regulate bacterial populations and can protect the epithelium from bacterial infections, reduced abundance in some of these bacteriophages is associated with increasing disease severity and a higher exacerbation rate (49). There is also a reduction in phage-bacteria pairs in asthma, suggesting that a reduced phage presence may contribute to microbial dysbiosis (50).

Children and adults with asthma display a distinct mycome when compared to healthy controls. The mycobiome of induced sputum of asthmatics is mainly composed of *Psathyrella candolleana, Termitomyces clypeatus and Grifola sordulenta which belong to the macromycetes (mushroom) family.* Asthma has been frequently associated with damp environments, but this is the first report of an association between macromycetes and asthma (51).

The mycobiome has also been related to asthma endotype, with Type 2-high asthmatics displaying a significantly lower fungal diversity that Type 2-low individuals. In particular, Alternaria, Aspergillus, Cladosporium, Fusarium, Penicillium, Trichoderma, Mycosphaerella and Wallemia are associated with atopy and Type 2 inflammation (51). Aspergillus is well known for inducing allergic presentations, and sensitisation is common in severe asthmatics. Additionally, Alternaria can induce airway sensitisation to allergens (52). Wallemia is directly correlated with sputum eosinophils, and Wallemia has been demonstrated to induce skin prick reactions and positive RAST testing in asthmatics (53, 54).

In addition, the mycombe in asthma is also influenced by inhaled corticosteroid (ICS) treatment. In a recent study, the authors were able to show an increased abundance of Ascomycota and reduced abundance of Basidiomycota 3 months post inhaled corticosteroid use. Interestingly, Basidiomycota is positively correlated with both sputum eosinophils and neutrophils (53). Increased burden of Aspergillus has also been documented post ICS treatment (55).

## Microbiome and asthma phenotypes

In asthma, clusters of clinical, demographical or pathophysiological characteristics are often referred to as phenotypes. In patients with severe asthma, phenotype-guided treatments are now available, predominantly targeting eosinophilic asthma. Clinical phenotypes include allergic asthma or atopic asthma, non-allergic asthma, late-onset asthma, asthma with persistent airflow limitation and asthma with obesity (1). Examination of the microbiome in various clinical phenotypes has revealed some taxonomic differences.

Clinical features in asthma, including airway hyper-responsiveness, degree of airway obstruction and level of symptomology have all been related to changes in the microbiome. Asthmatics with evidence of airway obstruction have a microbiome with lower alpha diversity and reduced abundance of commensal bacteria such as Firmicutes, Bacteroidetes and Actinobacteria in BAL when compared to asthmatics with normal lung function (56). Additionally, enrichment of Proteobacteria on bronchial brushings are associated with greater bronchial hyper-responsiveness (45). A further study examined the relationship between the gut microbiome and fixed airway obstruction in asthmatics in a tropical climate (57). Here different bacterial composition discriminated between patients with reversible and fixed airway obstruction, where enrichment of Streptococcaceae, Enterococcus and Veillonellaceae was seem in the fixed airway obstruction group (57).

Given asthma is such a heterogenous disease, with many clinical phenotypes, an attempt has been made to phenotype severe asthma by microbial composition. One study examining severe asthmatics, utilized unsupervised clustering of sputum microbiome of Bray-Curtis β-diversity measures and identified 2 microbial clusters, Cluster 1 and Cluster 2. Individuals in Cluster 2 had worse lung function, evidence of neutrophilia in blood or sputum and were more likely to receive add-on asthma medications. Cluster 2 also had decreased microbial richness and diversity compared to Cluster 1. They demonstrated reduction in many oropharyngeal commensals, including *Veillonella*, *Prevotella*, *Rothia* and *Neisseria*. Deficiency of commensal bacteria may give pathogenic bacteria opportunities to grow, and the study did show an increase in *H. influenza*, *M. cararrhalis* and *S. pseudopneumoniae* in the Cluster 2 microbiome. Interestingly, both clusters showed stability over an 18 month follow. As a follow up, induced sputum from individuals with mild to moderate asthma was also assessed. They predominantly fell into the less severe, Cluster 1 group (58). This study suggests microbial assessment may be used to phenotype patients with severe asthma, and potentially identify individuals who would benefit from interventions targeting the respiratory microbiome.

Allergic asthma or asthma with atopy is the most broadly recognized asthma phenotype. Atopy is considered the genetic predisposition to allergy, exhibited by a raised IgE response to an environmental allergen. Individuals often have childhood onset asthma, and a concurrent personal or family history of other allergic diseases. They also usually display eosinophilic inflammation and respond well to inhaled corticosteroids (59). In one study, asthmatic subjects with and without features of atopy display differing microbiota on bronchial brushings. Atopy was defined as serologic evidence of sensitization to at least one aeroallergen, but other markers of eosinophilic inflammation were not specifically compared. Here they showed enrichment of *Prevotella*, *Actinomcyes* and *Lactobacillus* in atopic asthmatics and enrichment of *Aggregatibacter*, *Haemophilus* and *Actinobacteria* in asthmatics without atopy (47).

### The microbiome and asthma symptoms

Symptomology in asthma is often assessed using the Asthma Control Test (ACT), a validated, patient reported assessment of asthma symptoms (60). Increased bacterial burden, in particular Proteobacteria is associated with worsening symptoms, when assessed by the ACT. Improving symptoms are associated with Actinobacteria, which produce many anti-inflammatory metabolites. Additionally, they are the main group associated with FKBP5 expression, which is a marker of steroid responsiveness, perhaps reflecting their role in symptom response to treatment. It is likely the varying microbial composition by clinical features reflects worsening dysbiosis associated with increasing disease severity, although increasing symptoms is associated with increasing treatment, which may also influence the microbiome.

## Microbiome and asthma endotypes

Asthma is a chronic inflammatory disease, with two main endotypes: Type 2-high and Type 2-low inflammation. Type 2-high inflammation is driven by Type 2 activation, leading to production of IL-4, IL-5 and IL-13 in response to varying stimuli (e.g., allergens, viral infection). These cytokines activate and recruit airway eosinophils, stimulate IgE production, raise exhaled nitric oxide (FeNO) and ultimately lead to airway remodeling. Type 2-high asthma is predominantly cortico-steroid responsive. In recent years, several biologic therapies have been developed to manage asthma with Type 2-high inflammation, including anti-IgE therapy and anti-IL5 therapies among others, discussed below (61). While Type 2-high asthma represents about 50% of the asthmatic population, Type 2-low or non-eosinophilic asthma makes up the remaining 50%. Type 2-low asthma is less well defined but exhibit a predominantly neutrophilic inflammation, responds less well to corticosteroids and patients have less treatment modalities available to them (59). The relationship between asthma endotype and the respiratory microbiome has been examined, with varying results, albeit most studies examine different populations with differing sampling techniques.

Sputum and bronchial brushings microbiome of Type 2-high asthmatics has a significantly lower bacterial burden and diversity compared to Type 2-low. Type 2-low sputum and brushing are found to be enriched with *Heamophilus*, *Moraxella* and *Neisseria* (62). Additionally, increased bronchial biopsy eosinophils, a marker of Type 2 inflammation is associated with a lower bronchial bacterial burden (63). A possible explanation is the bactericidal activity of airway eosinophils in Type 2-high inflammation (64). Another factor maybe the influence of non-bacterial microbiota, mainly fungi. The role of the mycome in asthma has been discussed in detail, however atopic asthmatics with high Type 2-inflammation have a significantly lower fungal diversity compared to Type 2-low subjects, assessed via endobronchial brushings (65).

Eosinophils are known to be a large component of Type 2-high inflammation. In a prior study, comparing BAL samples of asthmatics to healthy controls, asthmatics were further stratified into those with high or low eosinophils. The high eosinophilic group had higher alpha-diversity with enrichment of *Halomonas* and *Aeribacillus*. Conversely the low eosinophilic group had enrichment of *Neisseria*, *Actinomyces* and *Rothia* (66). Additionally, *Firmicutes*, mainly *Streptococcus* and *Actinomycetaceae* have been found to be enriched in eosinophilic asthma (67). Similarly, FeNO or fraction exhaled Nitric Oxide is often used as a surrogate marker for eosinophilic inflammation in asthma. A high FeNO has been shown to be associated with a lower prevalence of pathogenic organisms in sputum, including *Haemophilus* (68).

**Figure 1:**
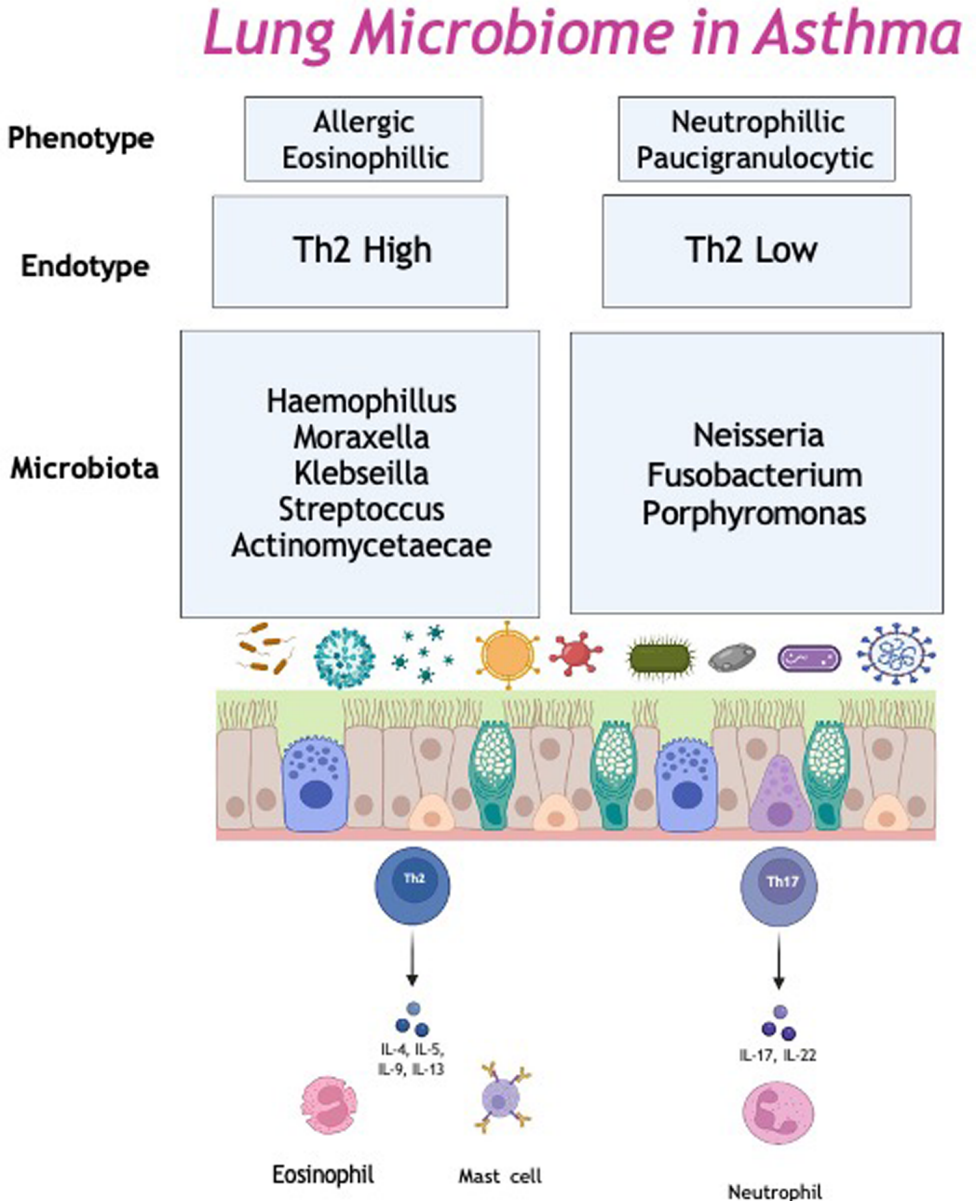
The lung microbiome in asthma. Th2, Type 2.

Another endotype; severe treatment resistant neutrophilic asthma, is associated with *M. catarrhalis*, *Heamophilus* and *Streptococcus* in sputum samples. Higher bacterial burden and the presence of gram-negative, potential pathogens may lead to increased bacterial cell wall fragments, such as lipopolysaccharides in the airway. Lipopolysacchardies bind to the pattern recognition receptors, triggering the innate immune response and initiate neutrophil recruitment (69). Thus, neutrophilic asthma is associated with excessive airway inflammation, with high levels of IL-1β, IL-6, IL-8, IL-12 and IL-17a in sputum. In particular, *H. influenzae* promotes a IL-17 mediated immune response, which has been show to induce a neutrophilic inflammation in a murine model of asthma. This suggests the increased bacterial burden leads to a Th17/IL-17 airway neutrophilic inflammation (70) (Figure 1).

With the abundance of evidence suggesting the role of the lung microbiome in the development, severity and heterogeneity of asthma it is reasonable to consider the microbiome as a specific target for manipulation in asthma prevention and/or treatment. The current treatment modalities in asthma include the use of bronchodilators, antibiotics, steroids, and more recently biologic therapy. Next we will review the contribution of the lung microbiome on specific asthma treatments.

## The effect of asthma treatment on the respiratory microbiome

Currently the management of asthma is guided by a symptom driven paradigm (71). Treatment historically focused on those who are sub-optimally controlled determined by symptom burden, exacerbation frequency and impact on quality of life. With this approach patients without symptoms but active disease may not be considered for targeted treatments. And although symptoms do not always correlate with the degree of airway inflammation, the degree of airway inflammation does predict serious adverse outcomes, risk of exacerbation and loss of lung function (72). More recently the introduction of novel biological therapy targets asthma phenotype. Most typically, eosinophilic airway inflammation in Type 2 high asthma, rather than symptom suppression and this has led to good biological and clinical response in carefully selected patients (73). Thus biomarker directed, phenotype specific management extends to include those with clinically demonstratable evidence of airway inflammation and airway dysfunction. Beyond this, current treatment guidelines do not address endotypes and therefore do not target the underlying pathophysiological process which would have the potential to prevent or reverse airway remodelling. This is a group that have the potential for disease modifying treatment before it manifests as a clinical challenge or fixed obstruction. Identifying therapies that include this cohort may reduce the risk that patients miss a therapeutic window with effective therapy and the microbiome may have this potential.

### Bronchodilators

Inhaled bronchodilators are a mainstay of asthma management, predominantly as reliver therapy. Bronchodilators can be used alone or in combination with inhaled corticosteroids (ICS) (71). Dual bronchodilators have a synergistic effect on the smooth muscle of the airway. β-2 adrenergic agonists and anticholinergics have a complementary effect on smooth muscle relaxation, reducing airway hyper-responsiveness and airflow limitation (74, 75). Historically, they have been considered predominantly as therapy for symptom relief. A common diagnostic test in asthma, bronchodilator responsiveness (or reversibility), entails an increase in FEV1 or FVC by 200mls or 12% post administration of a bronchodilator, according to the GINA guidelines (71). Unfortunately, a false positive test can occur in patients with an intercurrent infection (76). Serological evidence of a viral, Mycoplasmal or Chlamydial infection has been shown to induce a positive bronchodilator response in individuals with no known history of asthma and possibly contribute to overdiagnosis (76). In mini-pig models, when administered orally, salbutamol has been shown to effect gut microorganism characteristics (77). However, there is a paucity of studies investigating the effect of inhaled bronchodilators on the microbiome in asthma.

One study has examined microbial changes with formoterol, a long acting β-2 adrenergic agonist (LABA) in comparison to a LABA/ICS combination in subjects with COPD (78). While changes in the microbiome did not differ significantly, an unexpected increase in the alpha diversity was observed in the LABA monotherapy group, in essence, an increase in species richness and evenness as determined by the Shannon Diversity Index was observed. Although it is important to note that the combination group did demonstrate greater changes in the microbiome. Longitudinally these changes correlated with increased post bronchodilator FEV1 changes, suggesting changes to the microbiome may play a role in response to bronchodilator therapy. More studies are required to elucidate the exact role, if any, inhaled bronchodilators have on the microbiome in asthma, and the subsequent effect on underlying disease.

### Corticosteroids

Corticosteroids, both oral and inhaled make up the main component of asthma management, both chronically and during exacerbations. While research has established the benefit of ICS for both symptom control and reduction of airway inflammation (79), there is significant heterogeneity between studies looking at their effect on the respiratory microbiome. While limited, initial research suggests that they result in significant changes in bacterial presence. A systematic review signalled a trend towards increased diversity and altered composition (80). As detailed previously, asthmatic patients not established on ICS have a distinct microbial signature compared to those on maintenance treatment (81). The microbiome of steroid naive patients is enriched with *Haemophilus*, *Neisseria*, *Fusobacterium* and *Porphyromonas* (82). In those on ICS their microbiome reflects increased abundance of *Proteobacteria*. Interestingly, *Proteobacteria* has in turn been linked to neutrophilic asthma (38), which is generally not steroid responsive, indicating symptomatic treatment is not always in line with underlying inflammation and strengthening the need for personalised medicine (39).

The dose of ICS used also appears to influence the microbiome in stable asthma (83). In a prior publication, a lower ICS group was enriched with *Streptococcus*, and *Parvimonas* spp was enriched in the higher ICS group. Notably, the group receiving high dose fluticasone had a higher abundance of *H. parainfluenzae*, a common respiratory pathogen and also associated with corticosteroid resistance (82, 83). Some studies have suggested that ICS may increase pneumonia risk in asthma, in particular fluticasone, although the evidence is somewhat conflicting (84, 85). It may be that fluticasone exerts a selective effect on the respiratory microbiome, leading to overgrowth of pathogenic bacteria and subsequent clinical infection.

Oral corticosteroids also appear to influence the respiratory microbiome. A study compared endobronchial brushings in healthy controls not using corticosteroids, asthmatics on ICS alone and asthmatics on both ICS and OCS. Results indicated that OCS use significantly altered the relative abundance of various organisms within the microbiome, notably an increase in *Proteobacteria* and a reduction in *Bacteriodetes* and *Fusobacteria*. It was observed that changes to specific taxa were dose dependent, with a reduction in *Prevotella*, and increase Pseudomonas load with increasing OCS dose.

Several studies looking at corticosteroid responsiveness have demonstrated a correlation with the lung microbiome. Some research has been performed comparing the microbiota of patients who are categorised as steroid responsive or steroid resistant, supported by FEV1 measurements (82). In these studies, steroid-resistance individuals had expansions of gram negative organisms, including *Proteobacteria* when compared to those that were “steroid-responsive”. These organisms are predominantly lipopolysaccharide producing bacteria, suggesting possible microbial immune stimulation by LPS (82). Further investigation of one such organism, *H. parainfluenzae* in-vitro has shown that exposure to *H. parainfluenzae* in peripheral blood monocytes and BAL macrophages leads to a reduction in cellular response to corticosteroids (82). Moreover, lack of corticosteroid response by pulmonary macrophages is associated with airway inflammation in steroid resistant asthma (86). Notably, Proteobacteria and increased levels of LPS are associated with neutrophilic asthma, which again is classically steroid resistant (69). The composition of the lung microbiome may thus play a role in steroid-resistance and perpetrating underlying inflammation.

The majority of studies examining the association between corticosteroid use and the microbiome are observational, and it is difficult to establish if the changes in the microbiome are due to underlying disease severity or treatment affect. A longitudinal study assessed microbial changes in mild asthmatics following 6 weeks of ICS therapy. Again, *Haemophilus* was enriched in the non-responder group, but also, pre-treatment enrichment of *Heamophilus* was associated with steroid resistance. In this study, whole genome sequencing was used to assess microbial function. In this the authors found that those who did not respond to ICS had enhancement of xenobiotic degradation capacity, which may contribute to the lack of steroid response (47). However, a longer longitudinal study over 9 months found no difference between the induced sputum microbiome of those receiving and not receiving ICS, although different sampling methods may explain the varying results (53).

It appears cortico-steroid treatment, both inhaled and oral exerts an influence on the lung microbiome. The effect of oral corticosteroids on the GI microbiome in asthma has not yet been studied. Steroids may exert a selective effect on the microbiome, leading to dominance of pathogenic bacteria, and affecting airway inflammation, however longitudinal and metagenomic studies are sparse.

### Antibiotics

Long term antibiotics are now recommended for individuals with moderate to severe asthma, targeting those with frequent exacerbations, particularly macrolides with their anti-inflammatory immunomodulatory properties (87). The AMAZES trial demonstrated a significant reduction in asthma exacerbation rate and improved quality of life in patients with uncontrolled asthma post treatment with azithromycin (88). Despite clear evidence of benefit, its mode of action remains unclear. In addition to the above, the sputum microbiome was assessed as part of the AMAZES trail (89). Here, after 1 year of treatment, azithromycin reduced the abundance of *H. influenza*, without alterations in overall bacterial load, when compared to participants receiving a placebo. It also demonstrated an increase in the presence of antibiotic resistance genes, both macrolide and non-macrolide (89). A smaller study pre and post azithromycin therapy showed a reduction in airway pathogens, *Pseudomonas*, *Heamophilus* and *Staphylococcus*, suggesting an additional antimicrobial role of azithromycin (87). A further study examined the microbiome and metabolome post azithromycin treatment in individuals with COPD. Here the authors demonstrated a reduction in alpha diversity and notably an increase in airway bacterial metabolites, including glycolic acid, infole-3-acetate and linoleic acids, with a concurrent decrease in inflammatory cytokines. *Ex vivo* exposure of LPS-stimulated alveolar macrophages to the metabolites, but not azithromycin alone lead to a reduction in cytokine production. This suggests that it is azithromycin's effect on the microbiome, stimulating microbial production of anti-inflammatory metabolites that may drive its therapeutic benefit (90).

### Biological therapy

The introduction of monoclonal antibody biologic therapy in asthma over the last decade in particular has revolutionised treatment for those with severe refractory asthma. Currently available therapies target Type 2 cell-mediated pathways and are therefore licenced for use only in patients with evidence of allergic or non-allergic eosinophilic asthma (91). A new therapy, Tezepelumab is now indicated for non-T2 severe asthma, and hopefully will address this treatment gap. However, biologic treatment response can vary between patients despite comparable clinical and disease-specific biomarkers at treatment initiation (91). Therefore, there is a need to identify new treatable traits to improve biologic selection for severe asthmatic patients, thus aiding in predicting treatment response. Previous studies have shown that asthma phenotype, as well as eosinophil count and FeNo predict clinical response to these therapies (91). Furthermore, these asthma “biomarkers” have been associated with a distinct lung microbial signature such as microbial load, composition and diversity which, as discussed previously, have a role in asthma pathogenesis and treatment response. Therefore, it is possible that the lung microbiome has an important role in the biological therapy of asthma.

Omalizumab is an anti-immunoglobulin E (IgE) treatment currently used as add on therapy for severe allergic IgE mediated asthma. In a study of nine patients, bronchoalveolar lavage (BAL) sampling identified an altered microbial diversity following treatment with Omalizumab, with increased detection of *C. pneumonia*, *H. influenza*, *N meningitidis*, *Bordetella*, *S. pneumonia* and *S. aureus* and a decrease in H. influenza and M. pneumonia noted (92).

Mepolizumab is a humanised monoclonal antibody which targets interleukin 5 (IL-5), preventing binding to the IL-5R-alpha subunit on eosinophils. While it has proven efficacy in downregulating eosinophilic mediated local airway inflammation, an impact on the airway ecology is yet to be consistently demonstrated. In a group of 140 patients with severe asthma microbial composition before and after at least 3 months of mepolizumab was assessed. In this study, no significant change to the bacterial load or predominant organism of the airway microbiome in the post treatment subgroup was demonstrated (68).

Reslizumab is another IL-5 biologic therapy used in asthma. In a small study of 8 patients with severe asthma, microbial assessment of BAL microbiome via quantitative PCR was performed pre and 2 years post reslizumab treatment. In addition to a reduced BAL eosinophil count, the composition of the lung microbiota altered following treatment, with increased abundance in *Mycoplasma pneumonia*, *Neisseria meningitidis* and *Haemophilus influenza* (93).

Benralizumab is a biologic therapy that specifically targets the IL-5R receptor. To date there is little data on its impact on the lung microbiome in asthma. Interestingly, a recent publication evaluated the effect of benralizumab on the sputum microbiome in subjects with COPD. In this analysis, benralizumab lead to a reduction in bacterial load and specifically a reduction in *Streptococcus pneumoniae* when compared to placebo. However, this change in the microbiome did not correlate with lung function or symptomatology. Surprisingly, in this same manuscript, by *in vitro* analysis, the authors found that eosinophils were not involved in the clearance of *Streptococcus pneumoniae*, suggesting that benralizumab alters the bacteriome via a different pathway (94).

Importantly, biologic therapy is an established treatment option for refractory patients with Type 2 high asthma. However, there is little treatment options for patients with refractory Type 2 low asthma. In these patients there can be a predominant neutrophilic inflammation in the lower airways. Where seen, neutrophilic asthma is difficult to control and often steroid resistant (95, 96). Further research is clearly needed here. A better understanding of the complexity of the lung microbiome, and the functional capacity of micro-organisms may provide a potential therapeutic target, such as introducing a more favourable micro-environment, or even the eradication of certain taxa that may provide benefit in this cohort of patients that remain treatment elusive (97).

## Future directions

Recent interest in the microbiome as a key player in the pathogenesis, heterogeneity and severity of asthma will influence its role as a therapeutic agent. Areas where this may be of significant benefit and requires focus on future studies is Type 2 low and treatment resistant asthma where conventional treatment has not been successful in controlling symptoms. Correlation of clinical biomarkers and microbial signatures will allow for recognition of potential endotypes in a real world setting and thus facilitate targeted therapy. Further investigation into how the microbiome influences response or resistance to treatment is also needed. Research into whether the microbiome can be manipulated to increase response to established treatments will open up therapeutic options for difficult to treat asthmatics. For example, as discussed previously, although trials of probiotics for asthma prevention and management have shown mixed outcomes, they have demonstrated the potential for manipulating the microbiome to improve outcomes in asthma. Moreover, the microbiome varies with asthma phenotype, hence may be used to identify patients who will or will not benefit from certain treatments. This has the potential to prevent un-necessary interventions and streamline healthcare utilisation.

## Conclusion

Asthma is a complex disease with a range of phenotypes and endotypes. It is clear that the microbiome plays a key role in the development of asthma. Here we focus on the respiratory microbiome as it varies with disease severity, clinical phenotype and underlying inflammation. Established inhaled therapies used in asthma maintenance and treatment have been shown to alter the composition of the lung microbiome and may therefore explain both their efficacy and in some cases treatment resistance. Additionally, long-term antibiotics and oral cortico-steroids also exert an influence on the respiratory microbiome, suggesting their influence may be anti-inflammatory while also modulating the microbiome. Newer biological therapies have led to a significant paradigm shift in how we treat refractory Type 2 high asthma. While airway inflammation in this cohort of patients, historically represented by eosinophilic activity, was once the proposed mechanism of action of biological therapy, there is evidence to suggest that these treatments may also alter microbial signatures to create a more functionally balanced airway microbiome and reduce or modify airway inflammation directly. The downregulated inflammatory cascade seen clinically, biochemically and functionally supports the hypothesis that biological therapies affect the microbiome-host immune system and represent a potential and exciting therapeutic target for exacerbations and disease control.
